# Effect of Low-Frequency Repetitive Transcranial Magnetic Stimulation on Naming Abilities in Early-Stroke Aphasic Patients: A Prospective, Randomized, Double-Blind Sham-Controlled Study

**DOI:** 10.1100/2012/518568

**Published:** 2012-11-20

**Authors:** Konrad Waldowski, Joanna Seniów, Marcin Leśniak, Szczepan Iwański, Anna Członkowska

**Affiliations:** ^1^Second Department of Neurology, Institute of Psychiatry and Neurology, 9 Sobieskiego St., 02-957 Warsaw, Poland; ^2^Department of Experimental and Clinical Pharmacology, Medical University of Warsaw, 26/28 Krakowskie Przedmieście St., 00-927 Warsaw, Poland

## Abstract

*Background and Purpose*. Functional brain imaging studies with aphasia patients have shown increased cortical activation in the right hemisphere language homologues, which hypothetically may represent a maladaptive strategy that interferes with aphasia recovery. The aim of this study was to investigate whether low-frequency repetitive transcranial magnetic stimulation (rTMS) over the Broca's homologues in combination with speech/language therapy improves naming in early-stroke aphasia patients. *Methods*. 26 right-handed aphasic patients in the early stage (up to 12 weeks) of a first-ever left hemisphere ischemic stroke were randomized to receive speech and language therapy combined with real or sham rTMS. Prior to each 45-minute therapeutic session (15 sessions, 5 days a week), 30 minutes of 1-Hz rTMS was applied. Outcome measures were obtained at baseline, immediately after 3 weeks of experimental treatment and 15 weeks; posttreatment using the Computerized Picture Naming Test. *Results*. Although both groups significantly improved their naming abilities after treatment, no significant differences were noted between the rTMS and sham stimulation groups. The additional analyses have revealed that the rTMS subgroup with a lesion including the anterior part of language area showed greater improvement primarily in naming reaction time 15 weeks after completion of the therapeutic treatment. Improvement was also demonstrated in functional communication abilities. *Conclusions*. Inhibitory rTMS of the unaffected right inferior frontal gyrus area in combination with speech and language therapy cannot be assumed as an effective method for all poststroke aphasia patients. The treatment seems to be beneficial for patients with frontal language area damage, mostly in the distant time after finishing rTMS procedure.

## 1. Introduction 

The symptoms after brain damage are due as much to the changes in activity across the undamaged brain as to the actual damage [1]. Previous work speculates that the change in excitability in adjacent and contralateral homotopic regions of a cortical lesion is a consequence of reduced collateral (i.e., in ipsilateral perilesional regions) and transcallosal (i.e., in contralateral homotopic regions) inhibition [2–4]. This condition may interfere with plasticity processing during subacute phase of stroke thus makes worse the improvement of functions [5, 6].

Functional imaging studies after stroke in aphasics show increased activity in contralesional undamaged brain areas, but the role of these areas is controversial. Some activation in the uninjured brain could reflect adaptive cortical reorganization that promotes language recovery, but some changes (perhaps overactivation) may be maladaptive and generate the emergence of behaviors whose suppression would improve functional outcome. Nevertheless, normalizing the interhemispheric excitability within a bihemispheric language network and thus reactivation of perilesional areas has been suggested being most efficient in regaining language functions [7–10]. Even in chronic stroke patients, reengagement of primarily left language areas was demonstrated after speech and language therapy suggested them important for recovery of functions [11, 12].

Although some spontaneous recovery in the early period of stroke is due to a lesion-induced plasticity, the task for rehabilitation is to find ways of facilitating this plasticity so that the changes occur more rapidly and more completely. Repetitive transcranial magnetic stimulation (rTMS) is a noninvasive technique which may be favorable to stroke rehabilitation [13, 14]. rTMS is able to relatively normalize the neural activity in cortical areas of metabolic dysfunction. Depending on the stimulation parameters that are selected, it may have an excitatory or inhibitory effect on the neurons of the targeted brain area. High-frequency rTMS (>1 Hz) has been shown to transiently facilitate the neural activity. An advantage of inhibitory, low-frequency rTMS (≤1 Hz) is that rTMS modulates the level of excitability of a given cortical area beyond the duration of the rTMS train itself [15–18].

This study was designed to assess whether multiple stimulation of the right inferior frontal gyrus (RIFG) at 1 Hz frequency would produce significant language improvement in a group of early-stroke aphasia patients under double-blind, sham-controlled conditions. According to the model with three phases of language recovery after stroke proposed by Saur et al. [10], we hypothesized increased activation over the RIFG in all the subjects included to the study. By suppressing hypothetical maladaptive cortical plasticity in the region of interest and probably enhancing adaptive cortical activity in the left inferior gyrus (LIFG), we expected improvement in naming (accuracy and/or naming latency) in such patients. The current data suggests that naming abilities in aphasia are just related to the intensity of cortical activation in the left hemisphere, mostly in the left frontal areas [19, 20]. A growing number of studies have utilized these modulatory effects of rTMS on cortical excitability as a potential therapeutic technique in poststroke aphasics [21–27].

 In order to make the most of rTMS after-effect on plasticity occurs to a complete extend, all patients participated in specific speech and language therapy immediately after brain stimulation procedure.

## 2. Material and Methods

### 2.1. Subject

26 right-handed (assessed by the Edinburgh Handedness Inventory; Oldfield, 1971) [28], subjects with early stroke-induced aphasia of varying type and severity participated in the experiment. All subjects were native Polish speaking, aged 38 to 77 (mean ± SD age, 61.2 ± 10.8 years, 13 females, 13 males).  Table 1 provides detailed demographical and clinical information of the participants. The inclusion criteria consisted of the following conditions: (1) a first-ever left-sided middle cerebral artery (MCA) stroke with the lesion site confirmed by computer tomography (CT); (2) a stroke interval between 2 and 12 weeks at study onset; (3) age <80 years; (4) aphasia recognized in neuropsychological assessment with severe to moderate language deficits (1–4 points on the 6-point Aphasia Severity Rating Scale—ASRS); (5) patients who were able to name five or more pictures in the Computerized Picture Naming Test (CPNT), and (6) written informed consent in cooperation with the speech therapist and relatives.

The exclusion criteria were as follows: (1) total aphasia with poor nonverbal communication skills (0 points according to the ASRS); (2) visionandhearingdisabilities that might interfere with diagnostic and therapeutic treatment; (3) medications altering the level of cortical excitability (e.g., antiepileptics, neuroleptics, or benzodiazepines); (4) a history of premorbid dementia, substance abuse, or any neuropsychiatric diseases; and (5) contraindications for rTMS according to the safety guidelines [29, 30].

The protocol was approved by the Bioethics Committee at the Institute of Psychiatry and Neurology in Warsaw.

### 2.2. Procedure

 The study was randomized, double-blind, and sham-controlled. Patients were randomly assigned (using a standard table of random numbers before the commencement of the trial) either to the group receiving additionally to speech/language therapy real inhibiting rTMS (experimental group-E) or sham rTMS (control group-C). Because the patients had never experienced rTMS, they did not know whether they were receiving real or sham rTMS. All participants, investigators (except the one who was responsible for rTMS application), clinicians, speech and language therapist, and the patients' primary-care physicians were blinded to patient assignment to real or sham rTMS.

 The therapeutic procedure consisted of rTMS sessions and specific language training. Immediately after finishing rTMS treatment, both the experimental and control participants underwent speech and language therapy sessions for 45 minutes that were conducted every morning from Monday to Friday for 3 weeks. The speech and language training mainly focused on the expression and comprehension of spoken language rather than written language. Because the time since the stroke was relatively short (<3 months), the rehabilitation program focused on specific training to stimulate various aspects of the language system (e.g., semantic, phonological, syntactic, or motor). The general nature of the therapy method was similar for all patients, but the level of difficulty and types of exercises differed depending on the patients' symptoms. 

 It should be noted that some patients also participated in physical therapy treatment for coexisting motor deficits. The speech/language and physical therapy sessions occurred on the same day, after the break with the speech therapy session always scheduled first.

Naming assessment (pictures correctly named and reaction time) was obtained 3 times: at baseline, immediately after 3 weeks of experimental treatment, and again 15 weeks after the end of the therapy (follow-up) using the special-designed Computerized Picture Naming Test (CPNT). In order to qualify for entry into the study, each patient had to be able to name a minimum of 5 pictures on the 20 items of the naming test. 

Assessment and language training was performed by the same speech and language therapist, who was not aware of the patient's group randomization. Therefore, any influence of the individual features of the therapist or differences in rehabilitation approach was minimized.

### 2.3. Transcranial Magnetic Stimulation

 rTMS was performed with a Magstim Rapid stimulator (Magstim Company, Whitland, UK) equipped with an air-cooled figure-of-eight coil (each loop 70 mm in diameter). The subjects were seated in a reclining chair that allowed them to keep their arms and hands relaxed with the head leaning on the head-rest to be sure that it was immobile during rTMS procedure. The coil was placed tangentially to the scalp over the right IFG (RIFG). Magnetic stimulation was applied at 90% of the resting motor threshold (RMT) at 1 Hz frequency. RMT was determined in each subject once, before treatment, and was defined as the minimum stimulus intensity able to elicit a motor evoked potential (MEP) of at least 50 mV in 5 or more of 10 consecutive stimulations [31]. MEP was recorded from the first dorsal interosseus muscle of the unaffected hand. The stimulation parameters were chosen according to current safety guidelines for rTMS [29, 30]. 

The sham stimulation condition was performed with an air-cooled sham coil that looks and sounds similar to the discharge of real TMS coil (Magstim Co., Ltd.). The sham coil was placed at the same site on the scalp and with the same stimulation parameters (e.g., time and frequency) used for the real rTMS procedure.

 Because the spatial resolution of TMS is limited [32], it is impossible to only target those brain regions which are critical for task performance and it is similarly impossible to target a brain region which is absolutely not involved. In order to maximize the inhibitory rTMS after-effect over the whole RIFG area, we stimulated two parts of Broca's area homologues: the anterior part (pars triangularis—PTr) and posterior part (pars opercularis—POp). To target the regions of interest precisely, we positioned the coil on the scalp according to the coordinates used by Gough et al. [33]. The anterior stimulation site was 2.5 cm posterior to the canthus along the canther-tragus line and 3 cm superior to this line; the posterior stimulation site was 4.5 cm posterior and 6 cm superior to the canther-tragus line. On each day of treatment, rTMS was applied for 30 min. (15 minutes over the PTr and 15 minutes over the Pop, resp.). All the treatments were administered by only one investigator who was not involved in aphasia assessment and therapy. 

### 2.4. Measurements

All test measurements were obtained 1-2 days before study initiation in all subjects by the same, experienced speech and language therapist who was blinded to the stimulation type delivered to the patients. 

#### 2.4.1. Computerized Picture Naming Test (*CPNT*)

 This test consisted of 20 pictures of items (nouns) allowed to measure precisely: the accuracy of naming (i.e., the number of pictures correctly named) and reaction time (RT). For each picture-naming session, subjects were asked to name pictures presented on a 17-inch monitor controlled by a personal computer as fast as possible. The set of pictures contained items from a variety of separate semantic categories (e.g., animals, plants, furniture, clothing, vehicle, and household articles) of varying word frequency and length, presented in the same order for each subject. In addition, a list of 5 items was used at the beginning of the assessment to train the subject to the task. Each picture was shown on the screen for 30s with a 5s interstimulus interval between pictures and was preceded by a fixation exclamation mark. Verbal responses were audio recorded and digitized with software. The responses were transcribed off-line and scored to determine precise accuracy and RT of naming. The visual waveform on the monitor was used to measure the RT from onset of the picture presentation to the onset of the correct name. The accuracy of naming was scored “1” for correct responses and “0” for errors. 

#### 2.4.2. Boston Diagnostic Aphasia Test (*BDAE*) [34]

 This multifactorial test that measures different language abilities was used to determine the type of aphasia in patients enrolled to the study. Evaluation was based on the Polish adaptation of the BDAE [35]. To limit the time required for the testing session, the main deficits of aphasia were assessed on three test domains: naming, repetition and auditory comprehension. The following subtests of the BDAE were selected.

- Visual Confrontation Naming, Words Retrieval, Verbal Fluency (*naming*).
- Repetition of Words, Repetition Phrases and Sentences (*repetition*).
- Word Discrimination, Body-part Identification, Commands Comprehension, Complex Ideational Material (*auditory comprehension*).

#### 2.4.3. Aphasia Severity Rating Scale (*ASRS*)

This six-point (0–5) aphasia severity scale from the BDAE provides reliable ratings of communication impairment using a rating scale on which 0 represents “no usable speech or auditory comprehension” and 5 represents “minimal discernible speech/language handicaps.” 

A sampling of the free conversation with a patient as well as a picture scene description from the BDAE was used to determine aphasia severity at baseline and 15 weeks after completion of the therapeutic treatment (follow-up measurement).

### 2.5. Statistical Analysis

Data analyses were performed with SPSS for Windows statistical software. Differences in categorical data were analyzed using the chi^2^ test. According to the type of distribution (assessed by the Kolmogorov-Smirnov test), Mann-Whitney *U* test was used to compare the average values of baseline characteristics of the two groups (E and C). The efficacy of the applied therapy was assessed using a Mann-Whitney *U* test, because the normality of variable distribution was not confirmed, by comparing the average scores of the two groups. To control for multiple comparisons, the Bonferroni correction was applied. Hence, when three comparisons were made, *α* was set at 0.017.

## 3. Results

All patients completed the therapeutic treatment and accompanying testing sessions and tolerated the rTMS well. No adverse effect related to the application of rTMS was observed. Both groups were balanced at baseline according to the severity of aphasia, time since onset, age, years of education, and hours of speech/language training from the posttreatment exam to the followup and days between posttreatment assessment and the follow-up (Table 1). There were no significant intergroup differences in Computerized Picture Naming Test scores at baseline. 

Although both groups improved their naming abilities during 3 weeks of treatment significantly (expected improvement due to spontaneous recovery supported by the training program) and continued to show improvement in the follow-up examination, there was no significant difference in average test scores between groups at any time. The E group showed minimally better scores in average RT immediately after rTMS treatment; however, only a statistical tendency was observed (*U* = 46, *P* = 0.048, after Bonferroni correction). At the 15-week followup assessment, an improvement in RT was still maintained, but the intergroup difference in scores was not significant (*U* = 50, *P* = 0.077) (Table 2).

### 3.1. Additional Analysis

On the grounds of brain CT performed on admission, all participants were divided into three subgroups depending on the lesion site: anterior language area, posterior language area, and the whole language area (anterior-posterior). 

Patients with anterior part of language area damage were additionally analyzed. Other subgroups of patients with posterior lesion site were too small to perform statistical analysis or draw definite conclusions.

**Results for Subgroups with a Lesion Including the Anterior Part of Language Area**

Patients included in this analysis had an anterior lesion (AL) of the language area (selectively) or an anterior-posterior language lesion (APL). Subjects from the E group and the C group were balanced at baseline in terms of the severity of aphasia, age, and years of education but not according to lesion extension. In the E group, there were five subjects with AL and three with APL. The C group included four subjects with AL and six with APL (Table 3).

Even more C group had significantly longer time since stroke incident than experimental (*U* = 16.5, *P* = 0.037). However, all patients were in subacute phase of stroke characterized as lesion-induced spontaneous plasticity period, so that, described difference should not be influential to acquired results. 

There were no significant differences between these two groups in mean total CPNT scores at baseline. All naming test scores improved significantly in the groups after therapy and in the follow-up examination (Table 4). 

Immediately after the 3-week treatment no significant differences between the groups were noted, but the patients treated with real rTMS had a somewhat greater reduction in reaction time in naming (*U* = 20, *P* = 0.076). A 15-week follow-up examination resulted in occurrence of significant differences between the groups in the abovementioned field (*U* = 13, *P* = 0.016). 

Although both of the groups improved in accuracy of naming significantly in each consecutive testing point, there was only a strong trend in E group toward a significant increase in naming 15 weeks after rTMS treatment compared with C group (*U* = 20, *P* = 0.024), but the differences were not significant after correction for multiple comparisons. 

**Figure 1:**
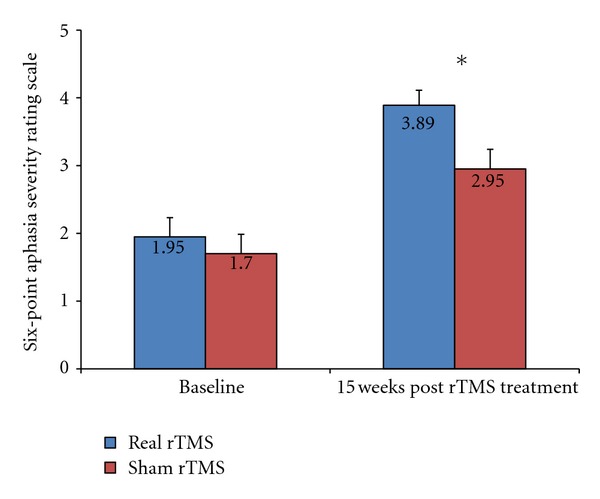
Subjects mean scores on the six-point Severity Rating Scale from the BDAE at baseline and 15 weeks post-treatment.

It is important to stress that there were significant differences between groups in ASRS ratings (defined in the study as the indicator of functional communication improvement) at the 15-week follow-up examination. Aphasia severity significantly decreased in the real rTMS group (*U* = 15, *P* = 0.021) compared to the sham group (Figure 1).

## 4. Discussion

 The results of the presented randomized, double-blind, placebo-controlled study did not confirm with the preliminary hypothesis that the application of low-frequency rTMS to the Broca's area right homologues improves significantly naming performance (accuracy and naming reaction time) in the early period of aphasia rehabilitation. 

Although both groups improved their language abilities during 3 weeks of treatment significantly and continued to show improvement in the follow-up examination, we cannot predict that visible improvement due to experimental procedure occurred. It is unquestionable, that in the early stage after stroke, the main mechanism of recovery is lesion-induced brain plasticity [36–38]. 

However, our data showed that the rTMS treatment seemed to have benefits for patients with a lesion that included or was located in the frontal part of the language area. Although the improvement was slightly visible after 3 weeks of rTMS treatment, it was more pronounced in advanced stage of stroke (a trend towards statistical significances in naming accuracy, but with significant decrease of reaction time at the follow-up examination).

Minimally better improvement in the experimental group might have been suggesting some favorable modulation effect of low-frequency rTMS on the frontal part of language network. 

We assume that these naming performance improvements after rTMS treatment, which likely suppressed the hypothetical overactivation of the RIFG in patients with early-stroke aphasia, probably occurred, at least in part, due to a shift in activation from the RH frontal areas to new activation in the LH perilesional areas. Accordingly, other studies have revealed a positive linear relationship between the intensity of LH activation, especially the left frontal cortex and naming abilities [20, 39, 40] during recovery from aphasia.

Our findings complement the results of Naeser et al. [22, 23], Hamilton et al. [24], and Barwood [25, 26], who observed improvements in naming abilities after inhibition of the right homologue of Broca's area in chronic nonfluent poststroke aphasics. The difference about our study is the targeting of the early-stroke patient cohort, who were administrated rTMS procedure in combination with speech and language training. An improvement in naming performance subsequent to low-frequency rTMS in combination with speech/language training in early-stroke aphasia patients was also observed in the study of Weiduschat et al. [27]. Finally, we enrolled 26 patients to our randomized, double-blind, sham-controlled study, while the previous studies were based on small samples or case studies. 

The naming improvement obtained by the experimental group of patients with the lesion including the anterior part of the language area compared to the control group was more visible over time, 15 weeks after finishing the treatment. It can suggest that rTMS may lead to starting an activation changes cascade within bihemispheric language neural network, which probably makes neuroplasticity more complete along time [24–26]. Time after stimulation may be a crucial factor in the interpretation of the effects of inhibitory rTMS on language processing in the present study. This is consistent with the previous studies which show that slow functional changes induced by inhibitory rTMS may occur over a period of months or years as reported in the fMRI study demonstrating altered patterns at 3 months after stroke that continued up to 46 months post-stimulation [41]. 

Our results additionally show that inhibiting the right Broca's homologue by rTMS in aphasia patients even if does not improve language functions significantly does not interfere with them. The absence of any negative rTMS effects on naming performance abilities suggests that the hypothetical overactivation presented to homologues of the lesion of the dominant hemisphere does not contribute to language performance in the early period of stroke and probably is not involved in rebuilding language network.

 Some of the previous studies generalized language improvement following contralesional 1-HZ rTMS stimulation of Broca's area beyond naming to other abilities, such as functional verbal communication skills [24, 41]. These results are consistent with our finding that patients who received real rTMS had significantly decreased aphasia severity, which was measured by a sample of free discussion between the therapist and the patient during the description of a picture scene. This data may provide evidence that rTMS induced a leftward shift of activation. Confirmation of this hypothesis can be found in the results of Ohyama et al. [42], who showed that the recruitment of the undamaged area surrounding the lesion within the posterior inferior frontal language area for spontaneous speech in aphasic patients was shown.

However, there might not be a causal link between improvement and the effect of rTMS. Unfortunately, we cannot say whether our 1-Hz rTMS protocol truly inhibited the RIFG in our patients and restored a shift in activation from RH frontal areas to new activation in LH perilesional, perisylvian areas. We hypothesize that this occurred, but we have no pre-rTMS and post-rTMS fMRI comparisons. Functional MRI comparisons would be critical for future research to understand the effects of rTMS on the activation levels in specific regions of interest, both local and remote from the site of rTMS. 

## 5. Conclusions

Low-frequency rTMS applied to the right frontal language homologue cannot be assumed as an effective method for all poststroke aphasia patients. It might be beneficial for selected patients (with a lesion including the anterior part of language area), but it needs to be confirmed in a larger series by controlled studies with a more homogeneous group of patients and longer followup. Further investigations are necessary to find optimum parameters of effective rTMS in patients with aphasia.

## Figures and Tables

**Table 1:** Patients characteristics (*n* = 26).

	E group (*n* = 13)	C group (*n* = 13)	*P* value
Gender M/F (*n*)	6/7	7/6	0.70^a^
Age (mean, SD)	62.31 (11.03)	60.15 (10.58)	0.57^b^
Years of education (mean, SD)	13.23 (3.92)	11 (2.00)	0.13^b^
Days from onset to pretreatment exam (mean, SD)	28.92 (19.39)	48.54 (32.33)	0.06^b^
Days from posttreatment exam to followup (mean, SD)	106.08 (8.62)	108.08 (6.30)	0.33^b^
Language training between posttreatment exam and followup, hours (mean, SD)	15.08 (13.71)	14.77 (15.04)	0.74^b^
Motor threshold (mean, SD)	56.85 (10.2)	59 (9.3)	0.57^b^
Aphasia severity—ASRS (mean, SD)	2.23 (1.01)	2.08 (1.4)	0.69^b^
Type of aphasia (*n*, %)			
Broca	3 (23.1)	3 (23.1)	^ c^
Wernicke	2 (30.8)	4 (15.4)	
Mixed	7 (38.5)	5 (53.8)	
Transcortical mixed	1 (7.7)	1 (7.7)	
Language lesion location (*n*, %)			
Anterior	5 (38.45)	4 (30.7)	^ c^
Posterior	5 (38.45)	3 (23.1)	
Anterior + Posterior	3 (23.1)	6 (46.2)	

E: experimental group, C: control group, SD: standard deviation, ^a^ *χ* ^2^, ^b^Mann-Whitney *U* test, ^c^analysis not performed due to small number of data.

**Table 2:** Mean total scores of naming test at baseline, after therapy and 15 weeks post-treatment.

	E (*n* = 13)		C (*n* = 13)	
	Accuracy	RT	Accuracy	RT
Pre-rTMS (mean, SD)	15.77 (4.76)	2.83 (1.55)	14.00 (5.77)	2.95 (1.54)
Immediately Post-rTMS (mean, SD)	17.92 (3.09)	2.07 (1.58)^†^	16.46 (4.56)	3.72 (2.76)
15-week followup (mean, SD)	19.31 (1.55)	1.99 (1.86)	17.62 (4.09)	3.04 (4.42)

E: experimental group, C: control group, SD: standard deviation, Accuracy: total number of pictures correctly named, RT: reaction time, *P*: level of significance in *U *Mann-Whitney test (*P < 0,017; ^†^ *P* < 0,05).

**Table 3:** Comparison of baseline characteristics between the two subgroups with lesion including the anterior part of language area.

	E group (*n* = 8)	C group (*n* = 10)	*P* value
Gender M/F (*n*)	4/4	5/5	1^a^
Age (mean, SD)	63 (12.07)	58.7 (11.38)	0.42^b^
Years of education (mean, SD)	13.3 (4.06)	11.1 (2.08)	0.28^b^
Days from onset to pretreatment exam (mean, SD)	26.6 (12.27)	55.4 (33.58)	0.037^b^
Days from posttreatment exam to followup (mean, SD)	106 (9.6)	108.1 (6.7)	0.76^b^
Language training between posttreatment exam and followup, hours (mean, SD)	18.25 (13.9)	14 (14.72)	0.3^b^
Motor threshold (mean, SD)	58.4 (11.8)	60.7 (12)	0.55^b^
Aphasia severity—ASRS (mean, SD)	2.5 (1.1)	2 (1.2)	0.34^b^
Language lesion location (*n*, %)			
Anterior	5 (38.5)	4 (30.7)	^ c^
Anterior + Posterior	3 (23.1)	6 (46.2)	

E: experimental group, C: control group, SD: standard deviation; ^a^ *χ* ^2^; ^b^Mann-Whitney *U* test; ^c^analysis not performed due to small number of data.

**Table 4:** Mean total scores of naming test at baseline, after therapy and 15 weeks post-treatment of the two subgroups with lesion including the anterior part of language area.

	E (*n* = 8)		C (*n* = 10)	
	Accuracy	RT	Accuracy	RT
Pre-rTMS (mean, SD)	15.88 (5.69)	2.45 (1.65)	14.70 (6.33)	2.32 (0.84)
Immediately Post-rTMS (mean, SD)	18.88 (2.23)	1.51 (0.65)	16.30 (4.83)	3.28 (2.27)
15-week followup (mean, SD)	20.00 (0.0)^†^	1.16 (0.41)*	17.70 (4.19)	2.93 (2.29)

E: experimental group, C: control group, SD: standard deviation, Accuracy: total number of pictures correctly named, RT: reaction time, *P*: level of significance in *U *Mann-Whitney test (*P < 0,017; ^†^ *P* < 0,05).
